# Response: Commentary: Morphologically Distinct *Escherichia coli* Bacteriophages Differ in Their Efficacy and Ability to Stimulate Cytokine Release *In Vitro*

**DOI:** 10.3389/fmicb.2016.01974

**Published:** 2016-12-09

**Authors:** Mohammadali Khan Mirzaei, Yeneneh Haileselassie, Marit Navis, Callum Cooper, Eva Sverremark-Ekström, Anders S. Nilsson

**Affiliations:** ^1^Department of Microbiology and Immunology, McGill UniversityMontreal, QC, Canada; ^2^Department of Molecular Biosciences, The Wenner-Gren Institute, Stockholm UniversityStockholm, Sweden

**Keywords:** pharmacokinetics, phage therapy, cytokines, immune response, multi-resistant bacteria

In their recent commentary on our manuscript entitled “Morphologically Distinct *Escherichia coli* Bacteriophages Differ in Their Efficacy and Ability to Stimulate Cytokine Release *In vitro*,” Dufour et al. suggest that the level of cytokine response generated in our paper is due to remaining bacterial debris rather than true differences between individual phages (Dufour et al., 2016).

Although at their core the two preparative methods are similar (both require filtration, CsCl centrifugation and dialysis). The additional purification step (the EndoTrap Blue system) binds endotoxins based on the conserved core of lipopolysaccharide (http://www.hyglos.de/en/products-services/products/endotoxin-removal/faq.html), which to our understanding is achieved through lipopolysaccharide specific binding to a phage derived protein. However, as Dufour et al. rightly point out, “bacterial lysates may also contain several pathogen-associated molecular patterns (PAMPs) able to elicit a pro-inflammatory response, such as flagellin (sensed by TLR-5), unmethylated CpG Oligodeoxynucleotide DNA (TLR-9), lipoteichoic acid from Gram-positive bacteria (TLR-2) and triacyl lipopeptides (TLR-1 with TLR-2) (Akira and Hemmi, 2003).” While such additional debris components may initially be in the minority of remaining debris, they would not be removed by the Endotrap system and may not be quantified as part of a Limulus amebocyte lystate (LAL) assay. Indeed, there have been previous instances where LAL assessment of phage preparations required alternative quantification methods (Merabishvili et al., 2009) or produced non-comparative results to an ELISA based system (Szermer-Olearnik and Boratyński, 2015). Therefore, had a true comparative analysis between the preparative methods and the same bacterial species been performed, differences could potentially have been less. However, to our knowledge no such studies have been performed.

With hindsight and a thorough data audit, we would certainly concur that at least some component of the observed cytokine responses are due to remaining contaminants and that additional purification steps such as chromatography will further reduce possible contaminants. However, such additional steps could come at a cost, reduce overall phage concentration and potentially require additional concentration steps (Boratyński et al., 2004). As such we would suggest appropriate caution when interpreting our data and would highlight that the other data presented in the paper are consistent with the position that phages are not directly cytotoxic (Merabishvili et al., 2009; Chhibber et al., 2014; Henein et al., 2016).

However, while it is likely that some component of the cytokine response generated is due to remaining contaminants, additional variation could also be introduced through factors which are uncontrollable, such as genetic variation in Toll receptors between different donors (Netea et al., 2012).

Despite the suggestion to the contrary provided by the Dufour et al. commentary, the reduction and quantification of endotoxin in phage preparations remains an active area of research (Branston et al., 2015; Szermer-Olearnik and Boratyński, 2015) as more effective, less labor intensive, and perhaps most importantly for commercial development, more cost effective methods are sought. However, this highlights the disparity between preparative methods for phage assessment and once again shows the urgent need for standardized methods and acceptance criteria, similar to those employed in antibiotic and biocide development to be developed.

## Author contributions

MKM, YH, MN, CC, ES-E, and AN: Writing of the reply and approved it for publication.

### Conflict of interest statement

The authors declare that the research was conducted in the absence of any commercial or financial relationships that could be construed as a potential conflict of interest.
